# Species-time-area and phylogenetic-time-area relationships in tropical tree communities

**DOI:** 10.1002/ece3.526

**Published:** 2013-03-15

**Authors:** Nathan G Swenson, Xiangcheng Mi, W John Kress, Jill Thompson, María Uriarte, Jess K Zimmerman

**Affiliations:** 1 Plant Biology Department, Michigan State UniversityEast Lansing, Michigan, 48824; 2 State Key laboratory of Vegetation and Environmental Change, Institute of Botany, Chinese Academy of Sciences20 Nanxingcun, Xiangshan, Beijing, 100093, China; 3 Department of Botany, National Museum of Natural History, Smithsonian InstitutionWashington, DC, 20013; 4 Institute for Ecosystem Studies, University of Puerto RicoRio Piedras, Puerto Rico, 00781; 5 Centre for Ecology and HydrologyEdinburg, Bush Estate, Penicuik, Midlothian, EH26 0QB, Scotland, U.K; 6 Department of Ecology, Evolution and Environmental Biology, Columbia UniversityNew York, 10027, NJ

**Keywords:** Community ecology, phylogenetic diversity, scaling, species-time-area relationship

## Abstract

The species-area relationship (SAR) has proven to be one of the few strong generalities in ecology. The temporal analog of the SAR, the species-time relationship (STR), has received considerably less attention. Recent work primarily from the temperate zone has aimed to merge the SAR and the STR into a synthetic and unified species-time-area relationship (STAR) as originally envisioned by Preston (1960). Here we test this framework using two tropical tree communities and extend it by deriving a phylogenetic-time-area relationship (PTAR). The work finds some support for Preston's prediction that diversity-time relationships, both species and phylogenetic, are sensitive to the spatial scale of the sampling. Contrary to the Preston's predictions we find a decoupling of diversity-area and diversity-time relationships in both forests as the time period used to quantify the diversity-area relationship changes. In particular, diversity-area and diversity-time relationships are positively correlated using the initial census to quantify the diversity-area relationship, but weakly or even negatively correlated when using the most recent census. Thus, diversity-area relationships could forecast the temporal accumulation of biodiversity of the forests, but they failed to “back-cast” the temporal accumulation of biodiversity suggesting a decoupling of space and time.

## Introduction

Predicting and quantifying the distribution of biodiversity through space has been a central goal of ecologists. Patterns of species diversity along ecological gradients and at multiple spatial scales are routinely quantified and mechanistic hypotheses are tested. A few generalities or laws have emerged from this work – most predominant among them is perhaps the species-area relationship (SAR). The SAR describes the increase in species richness with the area sampled. The SAR has attracted the attention of ecologists for decades providing the foundation for large basic and applied literatures (e.g., Preston 1960; MacArthur and Wilson 1963, 1967; Conner and McCoy 1979; Palmer and White 1994; Rosenzweig 1995; Qian et al. 2007).

The SAR is often represented using the Arrhenius (1921) power function:


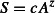


where *S* is the species richness, *A* is the area sampled, *c* is the intercept, and *Z* is the slope or the scaling exponent. A higher *Z* value indicates a greater increase in the number of species sampled with area. The *Z* value can therefore be used as an indicator of spatial heterogeneity in the species composition.

**Figure 1:**
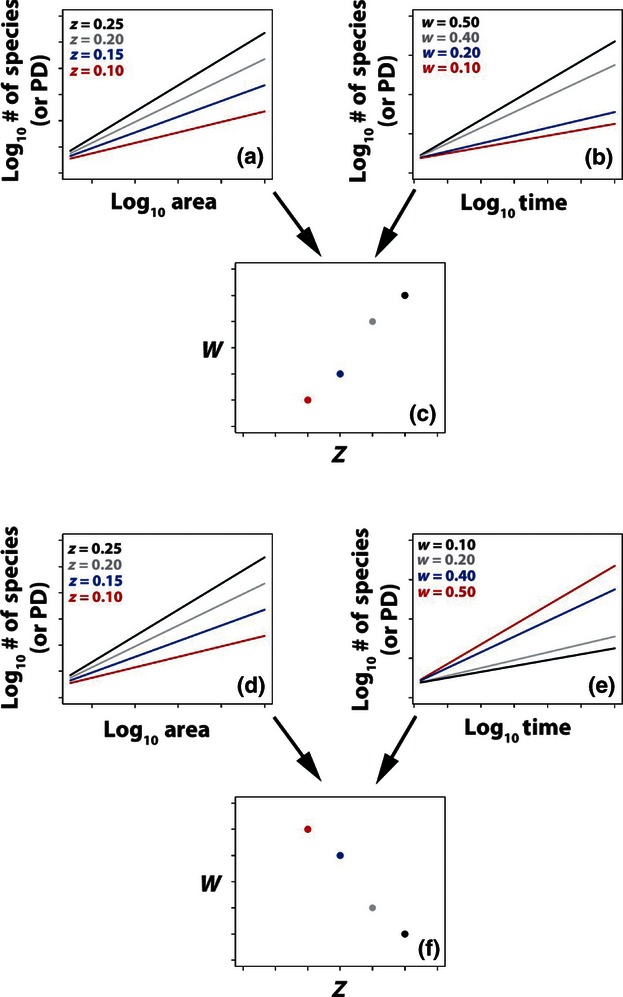
A conceptual figure representing the potential linkage between the species-area relationship (SAR) slope, *Z*, and the species-time relationship (STR) slope, *W*. In Panel a a *Z* is estimated for four different samples with the black community having the greatest accumulation of species through space and the red community having the fewest. In Panel b a *W* is estimated for the same four samples with the black community having the highest accumulation of species through time and the red community having the fewest. When comparing the *Z* and *W* values from these four samples in Panel c we find a strong positive correlation as predicted by Preston (1960). In Panel d a *Z* is estimated for four different samples with the black community having the greatest accumulation of species through space and the red community having the fewest. In Panel e a *W* is estimated for the same four samples with the red community having the highest accumulation of species through time and the black community having the fewest. When comparing the *Z* and *W* values from these four samples in Panel f we find a strong negative correlation thereby not supporting the prediction of Preston (1960).

Preston (1960) proposed a temporal analog of the SAR, the species-time relationship (STR), which describes the increase in species richness with the temporal duration of sampling. The STR can also be represented adequately using a power function (Adler and Laurenroth 2003; White et al. 2006):


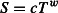


where *S* is the species richness, *T* is the temporal duration sampled, *c* is the intercept, and *W* is the slope or the scaling exponent. A higher *W* value indicates a greater increase in the number of species sampled with time. The *W* value can therefore be used as an indicator of temporal heterogeneity. Preston's original work suggests that spatial and temporal patterns of biodiversity should be closely linked, there should be an area-time interaction and that space and time may be substituted for one another. Specifically, the STR should be sensitive to the spatial scale at which the sampling is performed, the SAR should be sensitive to the temporal duration of the sampling and the scaling exponents from the SAR and STR of a community may be equivalent (Preston 1960). In other words, spatial and temporal heterogeneity should decrease with an increase in the temporal or spatial scale of the sampling and that *Z* should be equal to, or at least positively correlated with, *W* (Fig. 1).

Research into STR patterns has greatly lagged behind SAR investigations, but Preston's fundamental predictions regarding the interaction between SARs and STRs have formed the basis for a number of recent investigations. For example, Adler and Laurenroth (2003) have provided evidence supporting Preston's prediction that the STR scaling exponent decreases when sampling is carried out at larger spatial scales and that the SAR scaling exponent decreases when the temporal duration of sampling is increased. Further detailed empirical and simulation-based investigations have expanded this work to examine the interaction between area and time in plant communities (Adler 2004; Adler et al. 2005; McGlinn and Palmer 2009). Lastly, White et al. (2006) have shown that STRs are general across a broad variety of taxa. The recent burst in research into the species-area-time relationship (STAR) has highlighted its importance for understanding and predicting patterns of species diversity through space and time.

The recent work on the STAR has provided a solid foundation for linking spatial and temporal patterns of diversity. That said, there are many remaining avenues of research that can test and build upon this foundation. In this article, we highlight three such avenues. First, the STAR has generally been examined in temperate zone systems that are relatively species poor. For example, the STAR analysis of White et al. (2006), which represents the most geographically comprehensive analysis to date, included only one tropical locality, Hawaii, and the maximum diversity reported in any system was around 70 species. Interestingly, White et al. (2006) reported that the STR scaling exponent, *W*, was negatively related to species richness suggesting that future investigations into the STAR in diverse tropical communities would be insightful.

Second, the SAR and STR represent only one axis of biodiversity – species diversity. As species are evolutionarily non-independent and vary in their degree of similarity, alternative axes of biodiversity such as phylogenetic diversity (PD) can provide complementary or novel information critical to our understanding of the structure of communities (Faith 1992; Webb et al. 2002; McGill et al. 2006; Cavender-Bares et al. 2009). Therefore, expanding the STAR framework to include alternative axes of biodiversity should be a priority. Here we suggest that incorporating the phylogenetic component of biodiversity into the STAR framework is a natural progression as it is a biodiversity variable increasingly utilized in basic and applied ecological research (e.g., Webb 2000; Swenson et al. 2006, 2007). This can be accomplished by generating phylogenies representing regional pools and through the quantification of PD. The PD can then be substituted into the above SAR power function as:


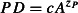


where the subscript P in the scaling exponent, *Z*, stands for phylogenetic. Recent work by Morlon et al. (2011) has also used a power function to successfully describe the phylogenetic-area relationship (PAR). Other work by Helmus and Ives (2012) has utilized an alternative function to compare pairwise PD and area. A pairwise PD measure is not considered here as it is not additive and therefore makes interpreting the PD relationship difficult.

The STR power function, as with the SAR power function, can be modified as:


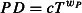


where the subscript P in the scaling exponent, *W*, again stands for phylogenetic. The *Z*_P_ and *W*_P_ therefore stand as alternative and perhaps complementary measures to the SAR and STR scaling exponents, *Z*_S_ and *W*_S_, which we now denote using the subscript S.

Third, the interaction between space and time suggests that space, in the form of the SAR scaling exponent, can be substituted for time, in the form of the STR scaling exponent. Thus, one may use spatial heterogeneity to predict the temporal heterogeneity and vice versa. This has not, to our knowledge, been directly tested by plotting *Z* against *W*. The expectation would be a strong positive correlation between these scaling exponents. Furthermore, we would expect that the temporal heterogeneity, *W*_S_ and *W*_P_, in both the future and the past to be predicted from the SAR scaling exponents, *Z*_S_ and *Z*_P_. Specifically the *Z* at time *t* should be positively correlated with the *W* and the *Z* at *t* *+* *x* should also be positively correlated with *W*.

The following work is designed to expand the STAR framework by examining the species-time-area and phylogenetic-time area relationships in two diverse tropical tree communities. First we expand the existing framework to incorporate the phylogenetic component of community biodiversity. Second, we test Preston's predictions regarding the interaction of the area sampled with the species-time and phylogenetic-time scaling exponents. Next we test for the existence of temporal decay in the species-area and PARs. Lastly, we ask whether the species-area and phylogenetic-area scaling exponents can predict both the past and future species-time and phylogenetic-time scaling exponents.

## Methods

### Forest dynamics plots

This study utilized two long-term tropical forest dynamics plots that share census protocols (Condit 1998). The first plot is the Barro Colorado Island (BCI) Forest Dynamics Plot located in central Panama (Hubbell and Foster 1983; Condit et al. 1996a). The BCI forest plot is a tropical lowland moist forest with an average annual rainfall of 2500 mm. The 50-ha plot was initially censused in 1982 and again in 1985, 1990, 1995, 2000, and 2005. During each census all free-standing woody stems greater than or equal to 1 cm diameter at breast height (1.3 m above the ground) are identified, measured and mapped. The BCI forest plot is primarily old growth forest with a small section having been disturbed.

The second forest plot was the Luquillo Forest Dynamics Plot (LFDP) located in the Luquillo Experimental Forest in Puerto Rico (Thompson et al. 2002, 2004; Brokaw et al. 2004). The Luquillo forest plot, on average, experiences 3500 mm of rainfall per year and is classified as a premontane tropical rain forest. The 16-ha Luquillo plot was initially censused in 1990 and again in 1995, 2000, and 2005. Large portions of the plot have a history of human land-use for agriculture and selective logging. The entire plot was also disturbed by Hurricane Hugo in 1989 and Hurricane Georges in 1998. Consequently the Luquillo forest plot is considerably more dynamic than old growth forest plots such as BCI providing opportunities to test this contrast.

### Inferring community phylogenies

A molecular community phylogeny was generated for both forest plots (Kress et al. 2009, 2010). Specifically, three commonly used plant DNA barcode regions (*rbcL*, *matK* and *trnH-psbA*) were sequenced and used to make a DNA supermatrix. The supermatrix was generated by globally aligning the *matK* and *rbcL* data and aligning the *trnH-psbA* sequence within families. The supermatrix and maximum likelihood were then used to infer a community phylogeny for each plot. Detailed methods regarding the extraction, sequencing, alignment, phylogenetic inference, and calibration can be found in Kress et al. (2009, 2010).

### Species-area and phylogenetic-area relationships

In each forest plot, a species-area and PRA was calculated for each 25 m^2^ subplot unless the subplot was within 50 m from the edge of the forest plots. This was done to avoid edge effects. The SAR for each subplot was quantified using a nested design where the species number in the 25 m^2^ subplot and the number of species in the 100 m^2^, 625 m^2^, 2500 m^2^, and 10,000 m^2^ surrounding the center of the subplot. The log species richness at each spatial scale was plotted against the log area for each subplot. A log-log relationship was compared to a semi-log relationship across all curves by comparing *R*^2^ values. On average, the log-log relationship fit the data better than a semi-log relationship. We therefore utilized the log-log relationship for all analyses where a regression was calculated for each subplot and the slope of the regression, *Z*_S_, was used to represent the SAR for that subplot. This procedure was repeated for each subplot in each census. The PAR, *Z*_P_, was quantified similarly except that instead of the number of species we utilized PD. PD is often represented as the proportion of the total phylogenetic tree length found in the focal sample thereby scaling PD between zero and one (Faith 1992). This work does not utilize this proportionality. Rather PD is defined here as the total phylogenetic branch lengths shared by the species found in a community.

### Species-time and phylogenetic-time relationships

The species-time and phylogenetic-time relationships (PTRs) were calculated using three different spatial scales – 25 m^2^, 100 m^2^, and 625 m^2^. Specifically we divided the forest plots into equally sized subplots at each spatial scale. Focal subplots within 50 m of the edge of the forest plots were eliminated from the analyses as these plots were not included in the species-area and phylogenetic-area calculations. In each subplot, we calculated the number of unique species at *t*_1_, *t*_1_ + *t*_2_, *t*_1_ + *t*_2_ + *t*_3_, etc. The log richness values were then plotted against the log time and a regression was calculated. As with the SAR and PAR relationships we compared log-log and semi-log plots and found that the *R*^2^ was on average higher for log-log relationships as has been reported in previous research on STRs (Adler and Laurenroth 2003; White et al. 2006). The slope of the log-log species-time regression, *W*_S_, was used to represent the STR for the focal subplot. This was repeated across all subplots at each of the three spatial scales. The PTR, WP, for each subplot was calculated similarly except that we calculated the accumulated PD (Faith 1992) instead of the accumulated number of species.

## Results

### Species-area and phylogenetic-area through time

**Figure 2:**
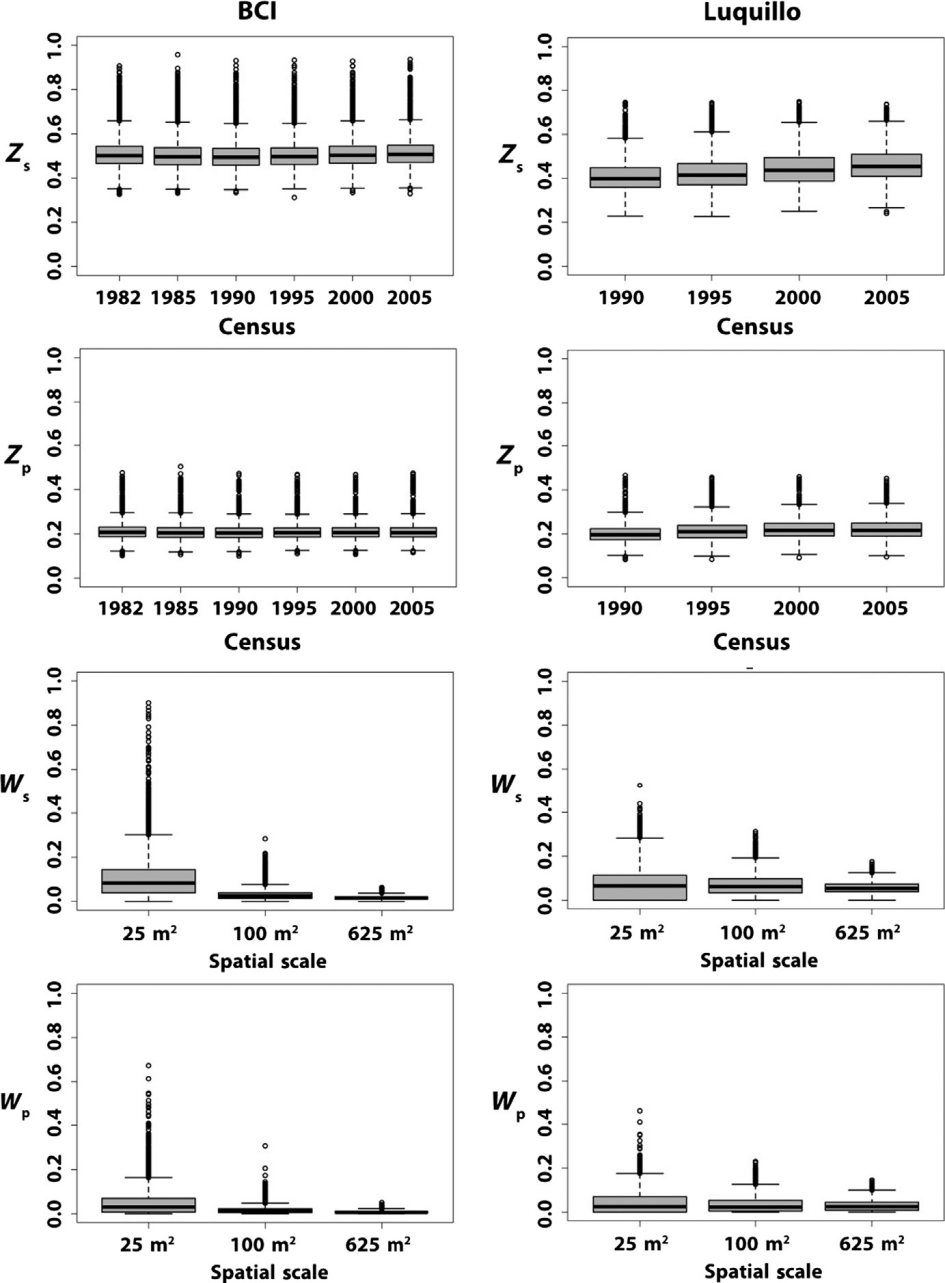
The top two rows display trends in the species-area relationship (SAR) slopes, *Z*_S_, and the phylogenetic-area relationship (PAR) slopes, *Z*_P_, across censuses in the Barro Colorado Island (BCI) and Luquillo forest dynamics plots (LFDP). The bottom two rows display trends in the species-time relationship (STR) slopes, *W*_S_, and the phylogenetic-time relationship (PTR) slopes, *Z*_P_, in the BCI and LFDP as the spatial scale of the sample increases. A Tukey test was used to determine significantly different distributions. Only the 25 m^2^ *W*_s_ and *W*_p_ values were significantly (*P* < 0.01) different from other spatial scales, while *Z*_s_ and *Z*_p_ values were indistinguishable through time.

The first goal of this study was to analyze trends in the species-area and PARs through time. This was done by quantifying the slope of the species-area and PARs for subplots within each forest plot. In general, the slopes of the species-area (*Z*_S_) and phylogenetic-area (*Z*_P_) relationships were higher in the BCI forest plot than that in the Luquillo forest plot (Fig. 2). The distribution of *Z*_S_ and *Z*_P_ at BCI was relatively constant through time, while the *Z*_S_ and *Z*_P_ values, on average, increased through time in the Luquillo plot (Fig. 2).

**Figure 3:**
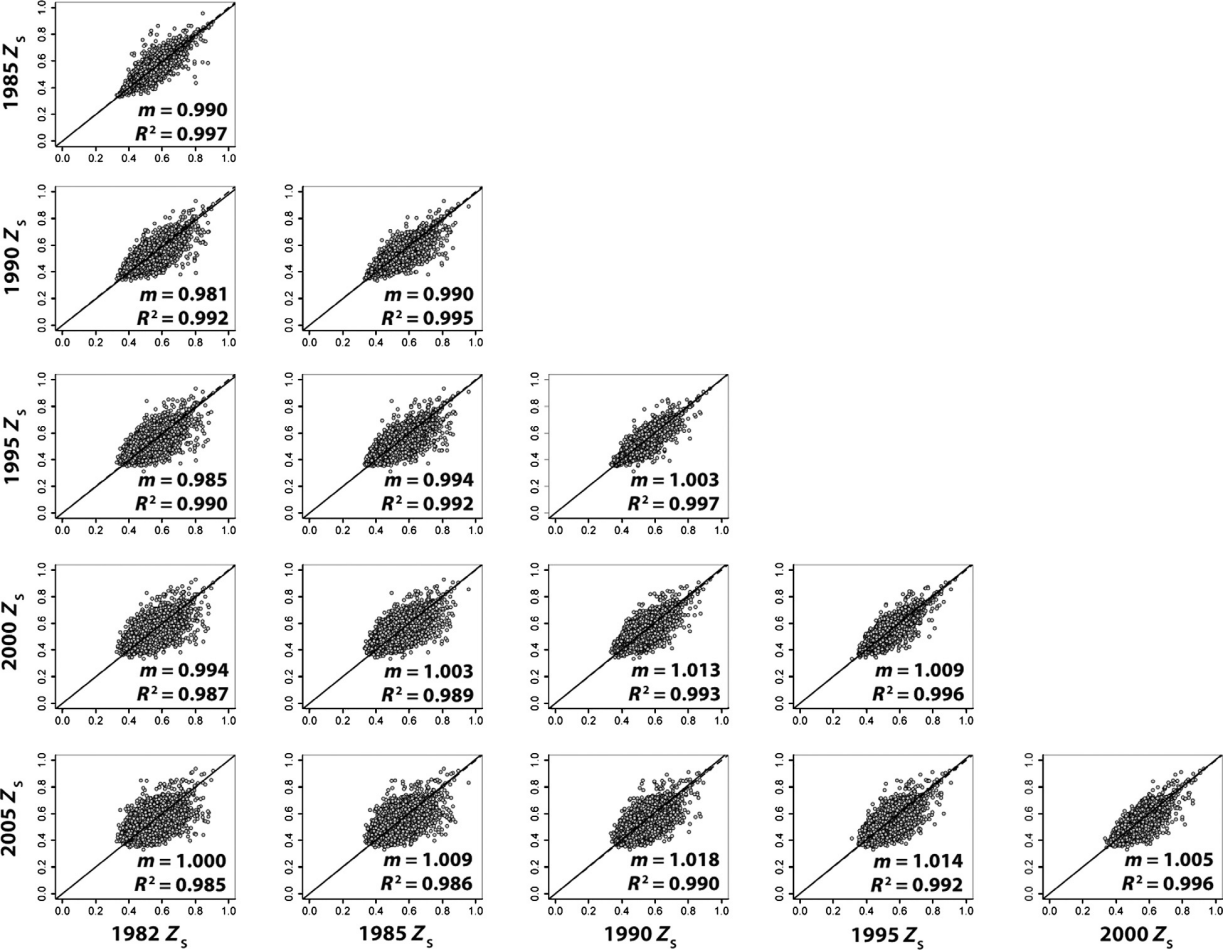
All pair-wise comparisons of the species-area relationship (SAR) slopes between censuses in the Barro Colorado Island BCI forest dynamics plot. The *R*^2^ and the slope of the regression through the origin are reported.

**Figure 4:**
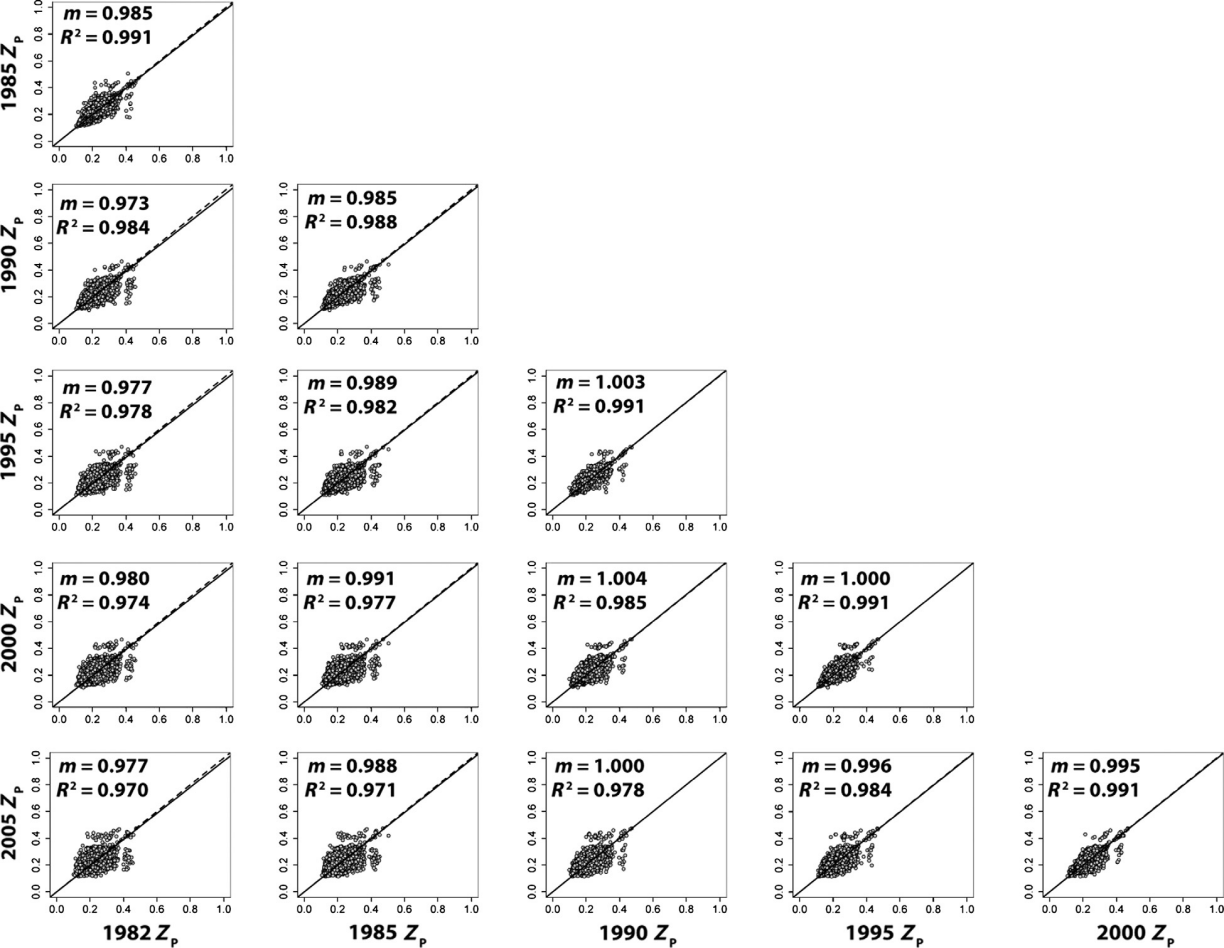
All pair-wise comparisons of the phylogenetic-area relationship (PAR) slopes between censuses in the Barro Colorado Island BCI forest dynamics plot. The *R*^2^ and the slope of the regression through the origin are reported.

**Figure 5:**
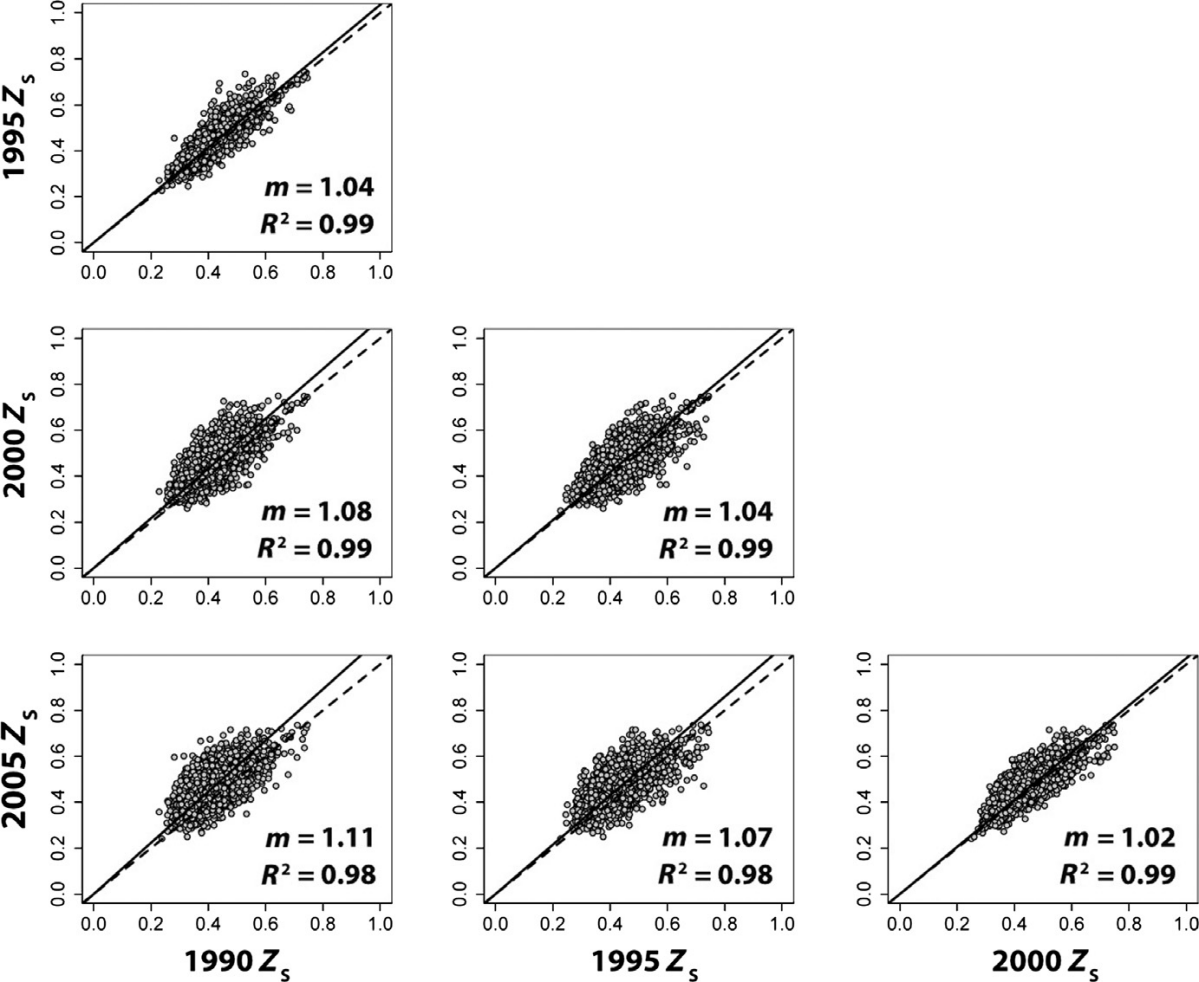
All pair-wise comparisons of the species-area relationship (SAR) slopes between censuses in the Luquillo forest dynamics plot. The *R*^2^ and the slope of the regression through the origin are reported.

**Figure 6:**
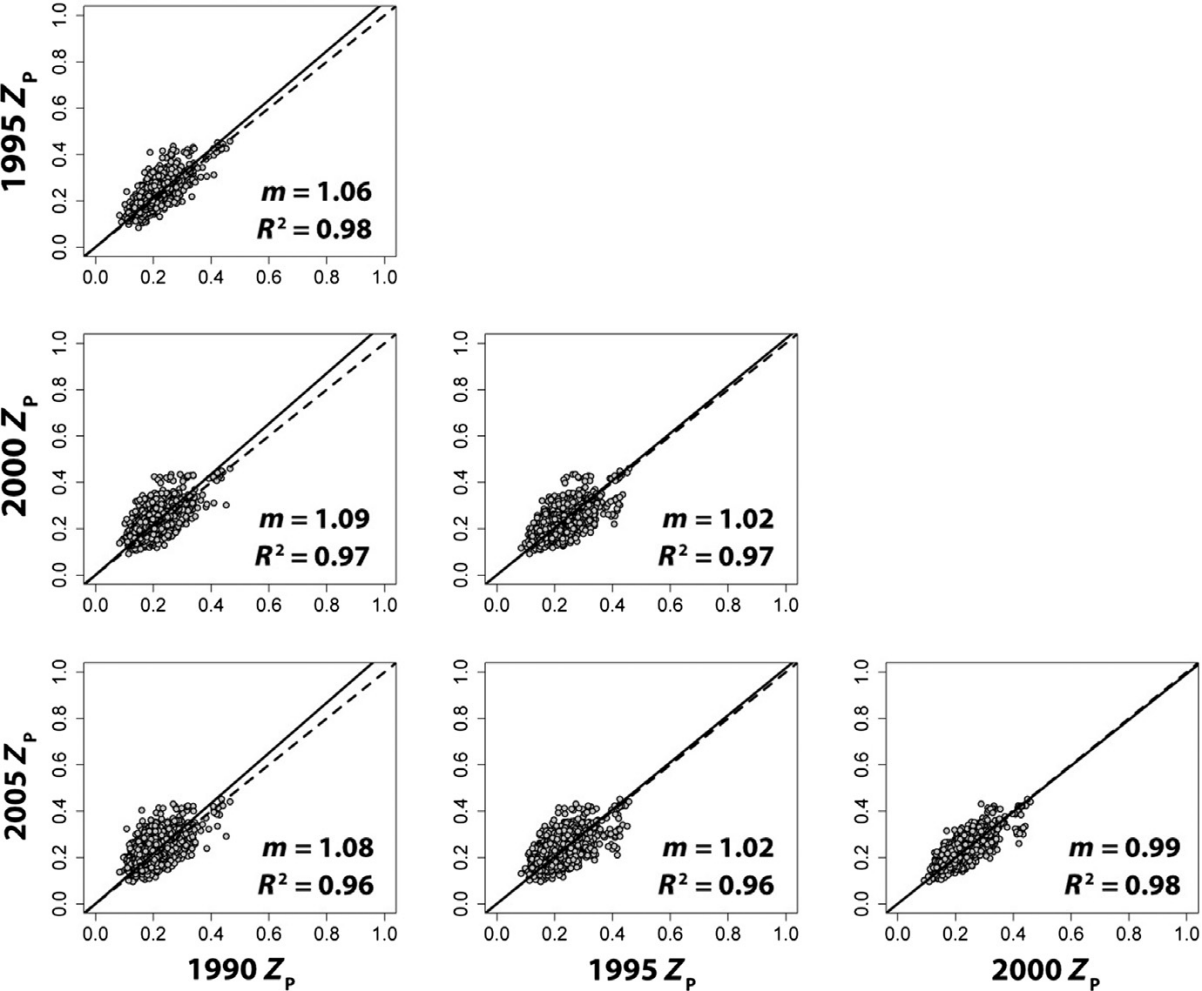
All pair-wise comparisons of the phylogenetic-area relationship (PAR) slopes between censuses in the Luquillo forest dynamics plot. The *R*^2^ and the slope of the regression through the origin are reported.

We also analyzed the temporal decay in the *Z*_S_ and *Z*_P_ values for particular subplots. Specifically, we regressed, through the origin, the *Z*_S_ and *Z*_P_ values for a subplot from one census against the values from all other censuses. At BCI the *Z*_S_ and *Z*_P_ values from one census were highly correlated (*R*^2^ > 0.970) with the values from all other censuses with regression slopes typically around unity (Figs. 3 and 4). That said, the strength of the correlation did decrease as the time between the censuses being compared increased. The *Z*_S_ and *Z*_P_ values for Luquillo were also highly correlated (*R*^2^ ≥ 0.960) across censuses, but nearly all of the slopes of the regressions were above unity suggesting an increase in spatial heterogeneity in the species and phylogenetic composition in Luquillo through time (Figs. 5 and 6).

### Species-time and phylogenetic-time across spatial scales

The second goal of this study was to analyze the relationship between spatial scale and the species-time (*W*_S_) and phylogenetic-time (*W*_P_) relationships. The variation in *W*_S_ and *W*_P_ values decreased as the spatial scale increased from 25 m^2^ to 625 m^2^ in both forest plots studied (Fig. 2). In the BCI forest plot the average *W*_S_ and *W*_P_ values significantly decreased as the spatial scale was increased from 25 m^2^ to 100 m^2^ to 625 m^2^ (Fig. 2). At Luquillo the *W*_S_ and *W*_P_ values, on average, between 25 m^2^ and 100 m^2^ spatial scales were not distinguishable, but they were found to be significantly larger than the *W*_S_ and *W*_P_ values calculated at a 625 m^2^ scale (Fig. 2). Thus, both the variance and mean *W*_S_ and *W*_P_ values were highest at small spatial scales in both forests suggesting the rate of species and phylogenetic temporal turnover tends to be highest on small scales.

### Area-time relationship

The last goal of this study was to quantify the correlation between species-area (*Z*_S_) and species-time (*W*_S_) relationships and the correlation between phylogenetic-area (*Z*_P_) and phylogenetic-time (*W*_P_) relationships. If spatial and temporal heterogeneity in the species and phylogenetic composition of tropical tree communities are positively associated, then the *Z*_S_ − *W*_S_ and *Z*_P_ − *W*_P_ relationships should be positive (Fig. 1). If spatial and temporal heterogeneity in the species and phylogenetic composition of tropical tree communities are negatively associated, then the *Z*_S_ − *W*_S_ and *Z*_P_ − *W*_P_ relationships should be negative (Fig. 1).

We quantified these correlations using *W*_S_ and *W*_P_ values calculated from all three spatial scales and *Z*_S_ and *Z*_P_ values calculated from all censuses. This allowed us to examine the species-time-area and phylogenetic-time-area relationships (PTARs) from multiple angles. In the BCI forest plot, the *Z*_S_ and *Z*_P_ values calculated from using the first and the second censuses were positively related with the *W*_S_ and *W*_P_ values, but these correlations were weak when using the largest spatial scale to calculate *W*_S_ or *W*_P_ (Tables 1 and 2). The *Z*_S_ and *Z*_P_ values calculated from later censuses were weakly positively correlated or, in some cases, negatively correlated with the *W*_S_ and *W*_P_ values.

**Table 1:** The Pearson's correlation between the species-area slope (*Z*_S_) and species-time slope (*W*_S_)

Forest plot	Census used for calculation of *Z*_S_	Area used for calculation of *W*_S_		

		25 m^2^	100 m^2^	625 m^2^
BCI	1982	**0.478**	**0.516**	**0.306**
	1985	**0.324**	**0.420**	**0.270**
	1990	0.029	**0.191**	**0.131**
	1995	**−0.103**	0.079	0.044
	2000	**−0.176**	0.003	0.006
	2005	**−0.227**	0.061	−0.029
Luquillo	1990	**0.146**	**0.137**	−0.027
	1995	−0.009	0.039	−0.032
	2000	**−0.297**	**−0.178**	**−0.131**
	2005	**−0.259**	**−0.159**	**−0.112**

Significant correlations are indicated in bold font. Positive correlations indicate a positive association between spatial and temporal heterogeneity. Negative correlations indicate a negative association between spatial and temporal heterogeneity (See Fig. 1).

**Table 2:** The Pearson's correlation between the phylogenetic-area slope (*Z*_P_) and phylogenetic-time slope (*W*_P_)

Forest plot	Census used for calculation of *Z*_P_	Area used for calculation of *W*_P_		

		25 m^2^	100 m^2^	625 m^2^
BCI	1982	**0.544**	**0.402**	**0.173**
	1985	**0.362**	**0.305**	**0.158**
	1990	0.099	**0.131**	0.086
	1995	−0.009	0.059	0.032
	2000	−0.064	0.016	0.021
	2005	**−0.110**	−0.029	−0.008
Luquillo	1990	**0.281**	**0.188**	0.007
	1995	0.042	0.050	<0.001
	2000	**−0.167**	−0.087	−0.009
	2005	**−0.161**	−0.064	0.006

Significant correlations are indicated in bold font. Positive correlations indicate a positive association between spatial and temporal heterogeneity. Negative correlations indicate a negative association between spatial and temporal heterogeneity (See Fig. 1).

The correlations calculated between *Z*_S_ and *W*_S_ and *Z*_P_ and *W*_P_ from the Luquillo forest plot were weaker than those found at BCI (Tables 1 and 2). Furthermore, a positive relationship was not found at the largest spatial scale. Lastly, mild negative correlations were recovered when comparing *Z*_S_ values from the two most recent censuses to the *W*_S_ values from those subplots. This suggests spatial homogeneity in later censuses occurring in areas that experience high species turnover or vice versa (Table 1).

## Discussion

The importance and utility of the interaction between space and time when investigating patterns of species diversity was first highlighted by Preston (1960). Preston's original framework made the fundamental predictions that the SAR should be sensitive to the temporal scale of the sampling and likewise that the STR should be sensitive to the spatial scale sampled. This work provided the foundation for recent investigations into what is now called the species-time-area relationship, or the STAR (Adler and Laurenroth 2003; Adler 2004; Adler et al. 2005; McGlinn and Palmer 2009). This work has generally supported Preston's predictions (Adler and Laurenroth 2003), but several questions remain. A recent broad scale analysis has suggested that the STR is sensitive to the species diversity of the ecosystem suggesting that investigations into highly diverse ecosystems would be informative (White et al. 2006). Furthermore, current research into the SAR and STR has generally only focused on the species diversity axes of biodiversity with less attention being paid to other axes such as PD (but see Morlon et al. 2011; Helmus and Ives 2012). Finally, the interaction between space and time outlined by Preston (1960) suggests that patterns of species or phylogenetic spatial turnover may be predictive of patterns of species or phylogenetic temporal turnover in the past and in the future. To our knowledge this prediction has not been explicitly tested. This article aimed to address these issues by analyzing the STAR and the PTAR in two tropical tree inventory plots.

As in previous studies, we also examined the influence of spatial scale on the STR, and now the PTR. In support of Preston's predictions and previous work from a temperate zone grassland (Adler and Laurenroth 2003) we found that as the spatial scale of the sampling increased the slope of the STR and PTR decreased (Fig. 2). Thus, the temporal accumulation of species is highest when sampling at fine spatial scales. This suggests that dispersal limitation on very local scales underlies an accumulation of species through time, but this accumulation is mitigated as the spatial scale of the analysis exceeds the dispersal kernel given the time period of sampling. This was true for both species-based measures and phylogenetically based measures of biodiversity. Interestingly, the slopes of the STR and PTR relationships reported here are substantially lower than those found in low diversity temperate ecosystems suggesting that the negative relationship between species diversity and the STR slope found by White et al. (2006) continues into the tropics.

We also tested to determine whether the slope of the SAR or PAR changed through time in the two tree communities studied. In the relatively older growth forest of BCI we found that the SAR slopes were generally consistent through time. Conversely, the recently disturbed Luquillo tree community had SAR and PAR slopes that significantly increased through time. This indicates that the forest became more spatially heterogeneous both in species and phylogenetic composition with time since disturbance. Previous work has suggested that priority effects may generate the reverse trend in temperate forests (Christensen and Peet 1984), but the results from Luquillo suggest a smaller or no role for priority effects in the initial composition of the forest following hurricane disturbance.

The consistency of the forest-wide SAR and PAR slopes in the BCI forest plot suggest that there may be no temporal decay, at least within the time frame of this study. Indeed the SAR and PAR slopes from each census were highly correlated, but the strength of the correlation did decrease with the time interval between censuses (Figs. 3 and 4). It is important to recall that the period of sampling is less than 30 years and dramatic changes in SARs over such a short period in an undisturbed forest may not be expected. That said, in the disturbed Luquillo plot the SAR and PAR slopes were also highly correlated with the relationship decaying slightly with time (Figs. 5 and 6). Furthermore, the steeper SAR and PAR slopes found in the later censuses caused the regression slope to exceed unity. This was expected given the results in Fig. 2 and again highlights the increase in the spatial heterogeneity in the species and phylogenetic composition at Luquillo with time since disturbance.

The final goal of this work was to determine whether the species or phylogenetic compositional heterogeneity in space is predictive of the temporal heterogeneity as proposed by Preston (1960). This prediction was tested by calculating the slope of the SAR or PAR at each census interval and comparing it to the STR or PTR slope calculated over all censuses. Preston's (1960) work originally suggested that the SAR and STR slopes may be equivalent thereby facilitating a space for time substitution or vice versa. The SAR and STR slope equivalency is predicated upon a precise sampling schema that is not followed in the tropical tree plots used in this study (Preston 1960). A further consideration is that a small number of censuses over a relatively short time period (∼20–30 years) are inadequate to estimate a STR slope. While these may be valid concerns and a longer term data would be preferable, it should be noted that over half of the original individuals in the 1982 BCI forest census are now dead (R. Condit pers. comm.) and far more than that have died in the Luquillo plot due to post hurricane dynamics (Swenson et al. 2012). Thus, an equivalency of SAR and STR, or PAR and PTR, slopes was not expected, but a significant positive relationship was still predicted to occur. Indeed when comparing the SAR and PAR slopes from the initial census of both forest plots to the STR and PTR slopes we found a significant positive correlation (Tables 1 and 2), but the strength of the correlation coefficients never exceeded 0.6. Thus, a large amount of variance was left unexplained even in the best scenario. The strength of the correlation further weakened as the spatial scale at which the STR and PTR slopes were quantified increased. The positive relationship between the SAR and STR slopes and the PAR and PTR slopes suggests that the initial spatial heterogeneity of the forests could have been used to roughly, though far from perfectly, estimate the future local scale temporal accumulation of species in these forests. This was true even in the more successional Luquillo forest plot, but we note that the correlation was much weaker (Tables 1 and 2). This suggests that disturbance may decouple the STAR as well as the PTAR.

The significant positive correlation between the SAR and PAR slopes of the initial census and the slope of the STR and the PTR provides some support for Preston's original prediction. However we again note that the strength of the relationship is not great. Interestingly, the positive correlation weakened or even reversed as later censuses were utilized to calculate the SAR or PAR (Tables 1 and 2). The negative correlation between the SAR or PAR slope calculated from the final census with the STR or PTR slopes indicates that areas with high species or phylogenetic accumulation through time are now more spatially homogeneous. We do note though that these negative correlations are weak and of more importance is the decoupling of the area-time relationship. Thus, we have found that the future temporal heterogeneity in these forests could have been roughly estimated using the initial measures of spatial heterogeneity, but we could not predict the previous temporal heterogeneity of the forests from present day patterns of spatial heterogeneity. In other words, temporal patterns could be forecasted, but not back-casted, from spatial patterns.

The space-time interaction in the species and phylogenetic compositions of the two tropical forests studied are more complex than originally envisioned by Preston (1960). The positive relationship between the initial spatial starting conditions and the future temporal turnover is in line with Preston (1960) and recent work (Adler and Laurenroth 2003; Adler 2004; Adler et al. 2005). This suggests that the present day spatial structure of these highly diverse tropical tree communities is somewhat informative when attempting to predict the future composition. This has important implications for both applied and basic research into tropical tree community ecology by highlighting the potential use of a space-time substitution. The nonexistent or weak negative relationship between the present day spatial structure and the past temporal accumulation of species was not expected and suggests that the space-time substitution or interaction is not as straightforward as was originally believed. In general, our results suggest that projecting backwards from present day spatial patterns is difficult, if not impossible, in the forests studied and that this was irrespective of the disturbance history of the forest.

## Conclusions

This work aimed to conceptually and empirically expand the growing literature on the STAR by considering the PTAR and by providing the first analyses of the STAR or PTAR in diverse tropical tree communities. We also aimed to test the original predictions of Preston (1960) regarding the interaction between spatial scale and the STR or PTR as well as the space for time substitution. Many of the results are congruent with Preston's original predictions and recent work from less diverse temperate zone systems. This suggests that the STAR translates into more diverse tropical ecosystems and to a phylogenetic framework. Future work may aim to extend the STAR and PTAR framework to incorporate functional diversity and a functional-time-area relationship and/or to investigate individual-area-time relationships (Condit et al. 1996b; Wiegand et al. 2007).

This work also highlighted areas where a space for time substitution in the tropical tree communities studied was more complex than that envisioned by Preston (1960) and that reported by recent investigations from temperate grasslands (e.g., Adler and Laurenroth 2003; McGlinn and Palmer 2009). Future empirical, theoretical, and simulation-based research will be needed to resolve why forecasting a STR or PTR is more tractable than back-casting it.

## References

[b1] Adler PB (2004). Neutral models fail to reproduce observed species-area and species- time relationships in Kansas grasslands. Ecology.

[b2] Adler PB, Laurenroth WK (2003). The power of time: spatiotemporal scaling of species diversity. Ecol. Lett.

[b3] Adler PB, White EP, Laurenroth WK, Kaufman DM, Rassweiler A, Rusak JA (2005). Evidence for a general species-time-area relationship. Ecology.

[b4] Arrhenius O (1921). Species and area. J. Ecol.

[b5] Brokaw NVL, Fraver S, Rear JS, Thompson J, Zimmerman JK, Waide RB, Losos EC, Leigh EG (2004). Disturbance history and canopy structure in two tropical forests. Tropical forest diversity and dynamism: findings from a large-scale plot network.

[b6] Cavender-Bares J, Kozak KH, Fine PVA, Kembel SW (2009). The merging of community ecology and phylogenetic biology. Ecol. Lett.

[b7] Christensen NL, Peet RK (1984). Convergence during secondary forest succession. J. Ecol.

[b8] Condit R (1998). Tropical forest census plots.

[b9] Condit R, Hubbell SP, Foster RB (1996a). Changes in tree species abundance in a Neotropical forest: impact of climate change. J. Trop. Ecol.

[b10] Condit R, Hubbell SP, LaFrankie JV, Sukumar R, Manokaran N, Foster RB (1996b). Species-area and species-individual relationships for tropical trees: a comparison of three 50-ha plots. J. Ecol.

[b11] Conner EF, McCoy ED (1979). The statistics and biology of the species-area relationship. Am. Nat.

[b12] Faith DP (1992). Conservation evaluation and phylogenetic diversity. Biol. Conserv.

[b13] Helmus MR, Ives AR (2012). Phylogenetic diversity-area curves. Ecology.

[b14] Hubbell SP, Foster RB, Sutton SL, Whitmore TC, Chadwick AC (1983). Diversity of canopy trees in a neotropical foerest and implications for conservation. Tropical rain forest: ecology and management.

[b15] Kress WJ, Erickson DL, Jones FA, Swenson NG, Perez R, Sanjur O (2009). Plant DNA barcodes and a community phylogeny of a tropical forest dynamics plot in Panama. Proc. Natl Acad. Sci. USA.

[b16] Kress WJ, Erickson DL, Swenson NG, Thompson J, Uriarte M, Zimmerman JK (2010). Improvements in the application of DNA barcodes in building a community phylogeny for tropical trees in a Puerto Rican forest dynamics plot. PLoS ONE.

[b17] MacArthur RH, Wilson EO (1963). An equilibrium theory of insular zoogeography. Evolution.

[b18] MacArthur RH, Wilson EO (1967). The theory of island biogeography.

[b19] McGill BJ, Enquist BJ, Weiher E, Westoby M (2006). Rebuilding community ecology from functional traits. Trends Ecol. Evol.

[b20] McGlinn DJ, Palmer MW (2009). Modeling the sampling effect in the species-time-area relationship. Ecology.

[b21] Morlon H, Schwilk DW, Bryant JA, Marquet PA, Rebelo AG, Tauss C (2011). Spatial patterns of phylogenetic diversity. Ecol. Lett.

[b22] Palmer MW, White PS (1994). Scale dependence and the species-area relationship. Am. Nat.

[b23] Preston FW (1960). Time and space and the variation of species. Ecology.

[b24] Qian H, Fridley JD, Palmer MW (2007). The latitudinal gradient of species-area relationships for vascular plants of North America. Am. Nat.

[b25] Rosenzweig ML (1995). Species diversity in space and time.

[b26] Swenson NG, Enquist BJ, Pither J, Thompson J, Zimmerman JK (2006). The problem and promise of scale dependency in community phylogenetics. Ecology.

[b27] Swenson NG, Enquist BJ, Thompson J, Zimmerman JK (2007). The influence of spatial and size scales on phylogenetic relatedness in tropical forest communities. Ecology.

[b28] Swenson NG, Stegen JC, Davies SJ, Erickson DL, Forero-Montana J, Hurlbert AH (2012). Temporal turnover in the composition of tropical tree communities: functional determinism and phylogenetic stochasticity. Ecology.

[b29] Thompson J, Brokaw NVL, Zimmerman JK, Waide RB, Everham EM, Lodge DJ (2002). Land use history, environment, and tree composition in a tropical forest. Ecol. Appl.

[b30] Thompson J, Brokaw NVL, Zimmerman JK, Waide RB, Everham EM, Schaefer DA, Losos EC, Leigh EG (2004). Luquillo forest dynamics plot, Puerto Rico, United States. Tropical forest diversity and dynamism: findings from a large-scale plot network.

[b31] Webb CO (2000). Exploring the phylogenetic structure of ecological communities: an example for rain forest trees. Am. Nat.

[b32] Webb CO, Ackerly DD, McPeek MA, Donoghue MJ (2002). Phylogenies and community ecology. Annu. Rev. Ecol. Syst.

[b33] White EP, Adler PB, Laurenroth WK, Gill RA, Greenberg D, Kaufman DM (2006). A comparison of the species-time relationship across ecosystems and taxonomic groups. Oikos.

[b34] Wiegand T, Gunatilleke CVS, Gunatilleke IAUN, Huth A (2007). How individual species structure diversity in tropical forests. Proc. Natl Acad. Sci. USA.

